# Single-molecule imaging of aquaporin-4 array dynamics in astrocytes†

**DOI:** 10.1039/d4nr00330f

**Published:** 2024-04-29

**Authors:** Anna-Lena Zepernick, Vanya Metodieva, Noelia Pelegrina-Hidalgo, Anna H. Lippert, Mathew H. Horrocks, Juan A. Varela

**Affiliations:** a School of Biology, University of St Andrews St Andrews UK; b EaStCHEM School of Chemistry, The University of Edinburgh Edinburgh UK; c IRR Chemistry Hub, Institute for Regeneration and Repair, The University of Edinburgh Edinburgh UK; d Center for Regenerative Medicine, The University of Edinburgh Edinburgh UK; e Institute for System Immunology, Julius-Maximilians-Universität Würzburg Würzburg Germany; f School of Physics and Astronomy, University of St Andrews St Andrews UK jv32@st-andrews.ac.uk

## Abstract

Aquaporin-4 (AQP4) facilitates water transport across astrocytic membranes in the brain, forming highly structured nanometric arrays. AQP4 has a central role in regulating cerebrospinal fluid (CSF) circulation and facilitating the clearance of solutes from the extracellular space of the brain. Adrenergic signaling has been shown to modulate the volume of the extracellular space of the brain *via* AQP4 localized at the end-feet of astrocytes, but the mechanisms by which AQP4 regulates CSF inflow and outflow in the brain remain elusive. Using advanced imaging techniques, including super-resolution microscopy and single-molecule tracking, we investigated the hypothesis that β-adrenergic receptor activation induces cellular changes that regulate AQP4 array size and mobility, thus influencing water transport in the brain. We report that the β-adrenergic agonist, isoproterenol hydrochloride, decreases AQP4 array size and enhances its membrane mobility, while hyperosmotic conditions induce the formation of larger, less mobile arrays. These findings reveal that AQP4 arrays are dynamic structures, responsive to adrenergic signals and osmotic changes, highlighting a novel regulatory mechanism of water transport in the brain. Our results provide insights into the molecular control of CSF circulation and extracellular brain space volume, laying the groundwork for understanding the relationship between astrocyte water transport, sleep physiology, and neurodegeneration.

## Abstract 2

Single-molecule imaging in astrocytes shows that aquaporin-4 array size and mobility are modulated by adrenergic signalling and tonicity changes. Created with BioRender.

## Graphical abstract

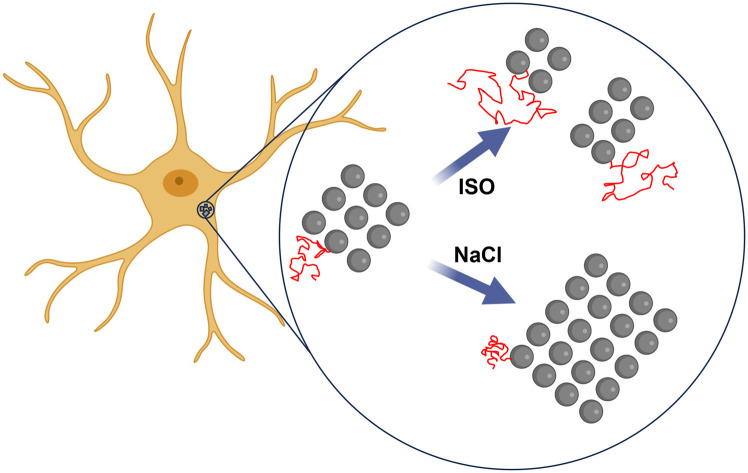

## Introduction

Aquaporins (AQP) are a family of proteins found in cell membranes which facilitate water transport into and out of the cell in accordance with the osmotic gradient. AQP4 is the most abundant water channel in astrocytes, highly expressed at their endfeet surrounding blood vessels. Electron microscopy experiments have shown that AQP4 can form large, strikingly regular arrays,^1–3^ but the physiological role of AQP4 arrays remains to be fully elucidated. It has been shown that the formation of arrays is essential for normal AQP4 expression levels,^4^ and the AQP4 isoforms M1 and M23 have opposing actions in regulating the size of the arrays.^5^ AQP4 arrays have been shown to diffuse at the plasma membrane of astrocytes,^6–8^ and they are stabilized at astrocytic endfeet by the dystrophin-associated complex,^9^ acting as intracellular scaffolding.^10^

AQP4 arrays at the endfeet of astrocytes have been shown to be crucial for the regulation of cerebrospinal fluid (CSF) circulation in the brain, being a central player in the so-called “glymphatic pathway” hypothesis. Mice lacking AQP4 showed reduced CSF inflow in the brain parenchyma and a significant reduction in extracellular solute clearance including amyloid beta aggregates.^11,12^ Although a causal link between AQP4 array mis-localization and neurodegenerative diseases has not been fully established, a reduction of endfeet localization of AQP4 in astrocytes has been correlated with the progression of Alzheimer disease in humans.^13^ It has been shown that the clearance of extracellular solutes in the brain is regulated by the sleep–wake cycle, correlated to a large change in the volume fraction of the extracellular space of the brain (ECS)^14^ and a change in the ionic composition of CSF.^15^ During sleep, or in conditions of general anesthesia, the ECS of mice enlarges by up to ∼60% compared to the wake state (or conditions of anesthesia with administration of adrenergic antagonists), thus enhancing waste removal.^14^ Adrenergic inhibition has also been shown to be neuroprotective after traumatic brain injury in mouse models, facilitating fluid drainage and reducing cerebral oedema.^16^ Similarly, adrenergic inhibition is also neuroprotective in models of ischemic stroke.^17^ Furthermore, it has been shown that in the rat visual cortex, the β-adrenergic receptor agonist isoproterenol hydrochloride (ISO) reduces ECS volume due to an expansion of astrocytic endfeet,^18^ but the mechanism behind this volume change remains largely unknown. Treatment with ISO has also been shown to affect astrocyte swelling in dissociated cell cultures, increasing intracellular cyclic adenosine monophosphate (cAMP) levels.^19,20^ Evidence from experiments done in the rat heart shows that ISO treatment reduces dystrophin levels,^21^ suggesting that a similar mechanism could happen in astrocytes, linking adrenergic signaling to AQP4 changes in localization. Further evidence for the role of AQP4 regulating astrocyte cell volume has arisen from studies of cytotoxic oedema formation,^22^ where AQP4 was shown to drive water down an osmotic gradient causing the astrocytes to swell.

Despite being such a fundamental player in CSF circulation and extracellular clearance, how AQP4 arrays in astrocytic endfeet regulate fluid circulation from the periarterial space to the parenchyma and subsequently to perivenous space remains unknown and has been a subject of debate since the glymphatic hypothesis was initially proposed. The main difficulty in studying the role of AQP4 in CSF regulation arises from the small spatial scales at which flow is regulated (nanoscale size and organization of arrays, submicron perivascular space size, thin astrocytic endfeet architecture and narrow gaps between endfeet).

Here, we use single-molecule imaging tools to test the hypothesis that activation of β-adrenergic receptors induce cellular changes that regulate AQP4 array size and mobility. Tuning the abundance of AQP4 at astrocytic endfeet will in turn regulate how much water is transported into an out of astrocytes, and subsequently into and out of the parenchyma. By imaging AQP4 at the nanometer length scale using direct stochastic optical reconstruction microscopy (dSTORM)^23^ in fixed cells, and through single-molecule tracking with quantum dots (QDs) in live cell conditions, we show that the adrenergic agonist ISO reduces the size of AQP4 arrays and increases their mobility in the plasma membrane. We further show that the opposite effect can be achieved by increasing the osmolarity of the medium, leading to larger and less mobile AQP4 arrays. These dynamic changes in AQP4 arrays suggest a new mechanism by which astrocytes regulate water transport, contributing to establishing functional links between sleep and wake cycle, and CSF flow in the brain.

### Adrenergic modulation of AQP4 array size

**Fig. 1:**
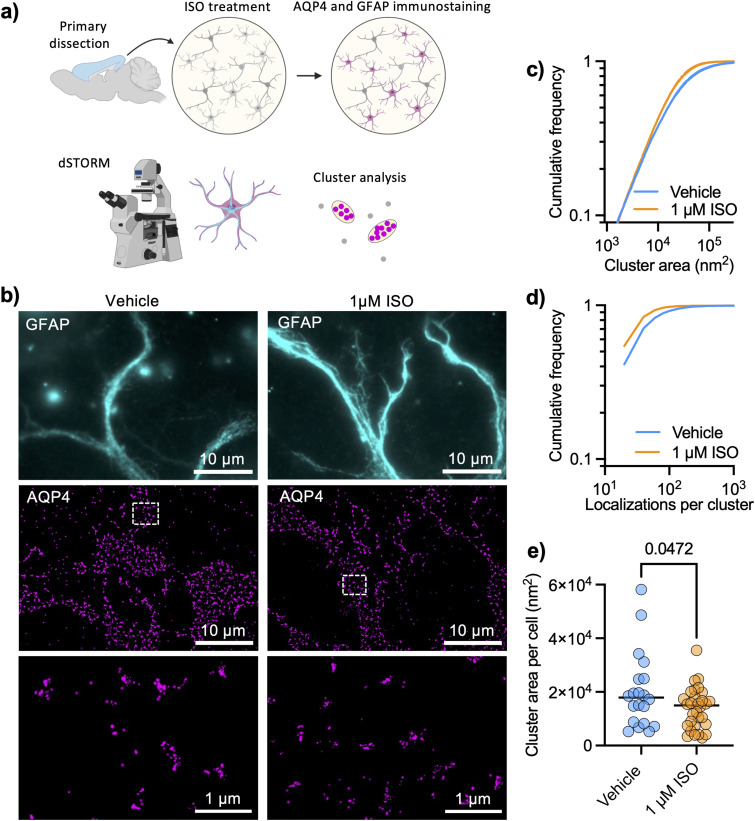
Super-resolution imaging reveals that adrenergic signaling regulates AQP4 array size. (a) Protocol illustrating dSTORM experiments to study AQP4 array size (created with BioRender). (b) Representative TIRFM images of cortical astrocytes stained for glial fibrillary acid protein (GFAP, cyan) with corresponding dSTORM images of AQP4 arrays (magenta) rendering localizations with dots of sizes corresponding to the respective localization precision. (c) Cumulative frequencies of the cluster size showing that 1 μM ISO significantly reduced cluster size after 1 hour (Kolmogorov–Smirnov test: *D* = 0.1085, *p* < 0.0001), (d) showing a reduction in the number of localizations per cluster (Kolmogorov–Smirnov test: *D* = 0.1481, *p* < 0.0001). Data obtained from three independent experiments with 20 fields of view (FOVs) and 16 161 clusters for vehicle conditions and 31 FOVs and 17 195 clusters for 1 μM ISO. (e) Cluster area per cell (median of distribution of each cell) shows a decrease in the cluster size upon ISO treatment (black lines show mean value; unpaired *t* test, *p* = 0.0472).

AQP4 arrays are highly structured and organized at a spatial scale that is well below the diffraction limit of light, so we chose a super-resolution imaging technique that is based on sequential detection of individual molecules in fixed cells.^24^ We performed dSTORM of AQP4 in DIV 9 cortical astrocytes (protocol illustrated in Fig. 1a) and examined how cluster sizes were affected by 1 μM ISO for 1 hour. The high localization precision of single-molecule imaging (ESI Fig. 1†) allowed us to analyze individual AQP4 arrays with the level of detail needed for such nano-structured objects (Fig. 1b), using glial fibrillary acidic protein (GFAP) to label intermediate filaments in astrocytes and facilitate cell identification. We found that the adrenergic agonist significantly decreased the size of detected clusters (Fig. 1c) in primary astrocytes, also significantly decreasing the number of localizations per cluster (Fig. 1d). The median cluster size in each cell also significantly decreased in size upon ISO treatment (Fig. 1e).

### AQP4 tracking in living astrocytes with functionalized QDs

**Fig. 2:**
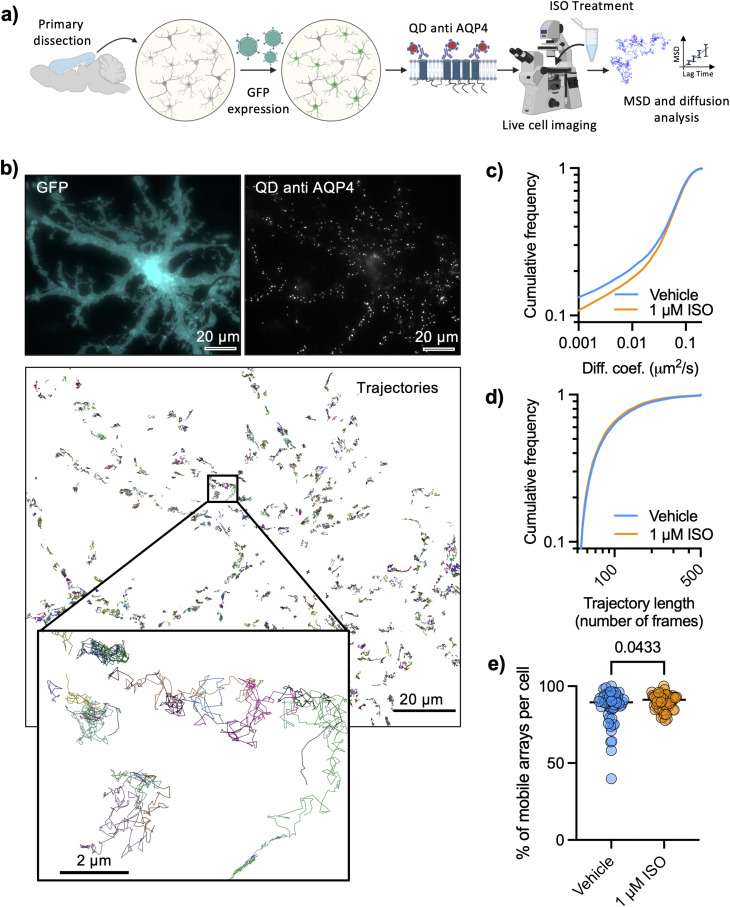
Adrenergic signaling tunes mobility of AQP4 arrays in living astrocytes. (a) Experimental workflow for quantum dot tracking (created with BioRender). (b) Representative images of cortical astrocytes expressing GFP, labeled with QDs functionalized against AQP4 with reconstructed QD trajectories. (c) Cumulative distributions of the diffusion coefficients of AQP4 (mean ± SD) in vehicle control conditions (*n* = 55 cells) and in response to 1 μM ISO (*n* = 56 cells). (d) Cumulative distributions of QD trajectory lengths (number of frames) for both experimental conditions. Diffusion data was obtained from five independent experiments (Kolmogorov–Smirnov test: *D* = 0.03431, *P* < 0.0001). (e) Percentage of mobile AQP4 arrays per cell for the same experiments shown in (c) and (d), showing an increase in the proportion of mobile *vs.* immobile arrays upon ISO treatment (unpaired *t* test, *p* = 0.0433).

To further evaluate the nanoscale regulation of AQP4 arrays by adrenergic signaling, we labeled AQP4 in living astrocytes using QDs functionalized with an antibody against the extracellular domain of AQP4 (Fig. 2a), at such low density of labeling that individual QDs could be identified and tracked for several seconds (Fig. 2b and ESI Movie†). Astrocytes were identified based on their morphology and the expression of GFP under astrocyte specific promotors (GFAP or GfaABC1D). The high specificity of AQP4 labeling with QDs could be seen by the enrichment of QD labelling on astrocytes displaying a very low labelling of non-astrocytic cells (ESI Fig. 2a†) as well as a very low labeling of QD coated with an off-target anti His tag antibody, showing that the number of QDs per μm^2^ was significantly lower (28-fold reduction) with the off-target antibody (ESI Fig. 2b†). Mobility of functionalized QD was quantified reconstructing their trajectories and calculating the mean-squared displacement, to subsequently calculate the diffusion coefficient of each trajectory with standard Brownian motion analysis tools.^25–27^ We carried out AQP4 tracking in living astrocytes that were pre-treated with 1 μM ISO for 10 minutes and observed a significant increase in mobility of tracked QD (Fig. 2c), compatible with the reduction in size of AQP4 arrays observed with dSTORM which would lead to a faster Brownian motion of the arrays. Both conditions showed equivalent distributions of trajectory lengths (number of time points per trajectory), suggesting that tracking protocols were able to correctly describe the diffusion of both experimental conditions (Fig. 2d). The fraction of mobile QD in each cell (diffusion larger than 0.001 μm^2^ s^−1^) significantly increased upon ISO treatment (Fig. 2e).

### AQP4 array modulation by osmotic changes

**Fig. 3:**
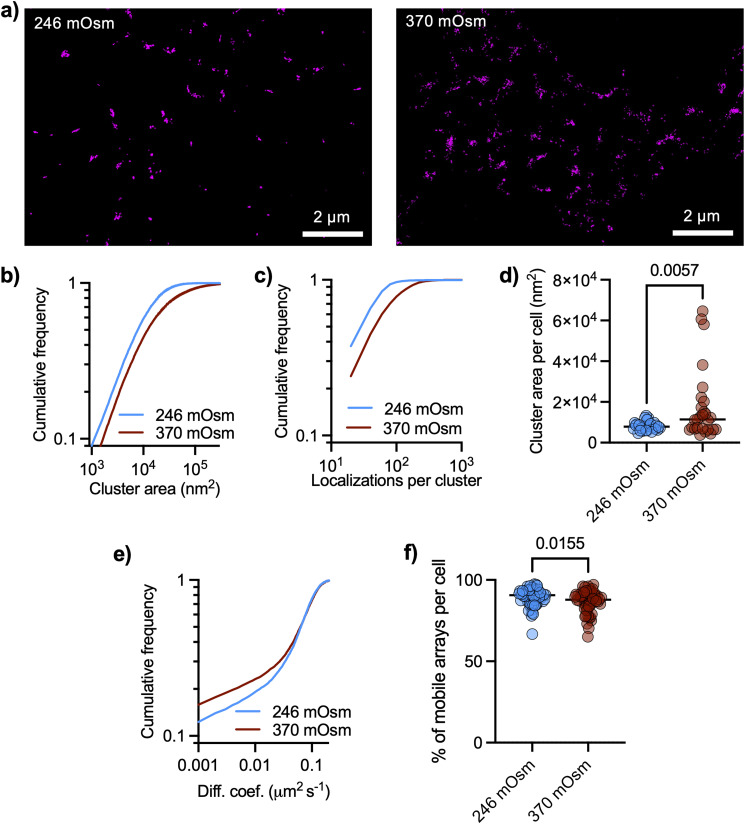
Osmolarity changes regulate size and mobility of AQP4 arrays. (a) Representative TIRFM image of DIV9 cortical astrocyte stained for GFAP (cyan) with corresponding dSTORM images of AQP4, rendering localizations with dots of sizes corresponding to the respective localization precision. (b) Cumulative frequencies of the cluster size show an overall increase in cluster sizes in hypertonic condition (Kolmogorov–Smirnov test: *D* = 0.1832, *P* < 0.0001). Data were obtained from three independent experiments with 28 FOVs with 17 894 clusters for isotonic medium (246 mOsm) and 27 FOVs with 25 977 clusters for hypertonic medium (370 mOsm). (c) Cumulative distribution of the number of localizations per cluster (Kolmogorov–Smirnov test: *D* = 0.2835, *p* < 0.0001). (d) Cluster area per cell (median of distribution of each cell) shows an increase in the cluster size upon hypertonic treatment (black lines show mean value, unpaired *t* test, *p* = 0.0057). (e) Cumulative distributions of the diffusion coefficients of AQP4 (mean ± SD) comparing isotonic *vs.* hypertonic conditions (Kolmogorov–Smirnov test: *D* = 0.03297, *P* < 0.0001). Data represent two independent culture replicates (isotonic *n* = 56 cells, hypertonic *n* = 56 cells). (f) Percentage of mobile AQP4 arrays per cell showing an decrease in the proportion of mobile *vs.* immobile arrays with hypertonic conditions (unpaired *t* test, *p* = 0.0155).

As AQP4 transport water across the plasma membrane driven by osmotic gradients, we asked if cluster size could be modulated by changing the tonicity of the culture medium. dSTORM images revealed distributed nanoclusters of AQP4, with cluster size significantly increased after raising the tonicity of the cell culture medium with sodium chloride from 246 mOsm to 370 mOsm (Fig. 3a and b), together with a significant increase in the number of localizations per cluster (Fig. 3c). The median cluster size in each cell significantly increased in hyperosmotic conditions (Fig. 3d). We also performed QD tracking of AQP4 in living astrocytes under these changes in medium osmolarity and found a significant slow-down of AQP4 arrays (Fig. 3e). The fraction of mobile QD in each cell (diffusion larger than 0.001 μm^2^ s^−1^) significantly decreased in hyperosmotic conditions (Fig. 3f). The slower Brownian diffusion of arrays in hyperosmotic conditions is consistent with the measured increase in array size.

We integrated data from QD tracking and dSTORM by double labeling AQP4 (intracellularly and extracellularly) to confirm that QD are able to label arrays. For this we used two different antibodies against AQP4, one of them binding intracellularly and the other extracellularly (showing similar staining patterns, ESI Fig. 3†). We labeled AQP4 with functionalized QD (extracellularly) on living astrocytes, subsequently fixing them with paraformaldehyde and used another antibody against AQP4 (binding intracellularly) to perform dSTORM with a secondary antibody labeled with Alexa Fluor 647 as illustrated in ESI Fig. 4a.† Astrocytes showed arrays labeled with both QD and dSTORM confirming that QDs can label AQP4 arrays (ESI Fig. 4b†). We further tested this by doing live cell imaging tracking experiments with a higher concentration of functionalized QD (see Materials and methods) and examined the fluorescent signal of individual spots (which could be single or multiple QD). Intensity profiles along trajectories show trajectories corresponding to both single and multiple QD, as some trajectories showed several well-defined levels of intensity and blinking patterns QD (*e.g.* ESI Fig. 5†).

## Discussion and conclusions

Using a combination of super-resolution and single-molecule imaging approaches, we tested the hypothesis that activation of β adrenergic receptors regulates AQP4 assembly, mobility, and expression. While previous published tracking experiments of AQP4 were done with engineered AQP4 in overexpressed systems with anti-myc tag antibodies,^6,8,28^ our single-molecule tracking based on QDs functionalized with an antibody against AQP4 was done in endogenous conditions, with very high levels of specificity. This allowed us to test how endogenous levels of AQP4 respond to external modulatory stimuli in primary astrocytes. Our data show, to our knowledge, the first evidence for a new regulatory mechanism of AQP4 array size and mobility in response to adrenergic signaling and osmotic changes. Previous electron microscopy studies revealed that AQP4 forms square arrays, whose assembly may be regulated by opposing actions of the two splice variants, with M23 composing large arrays and M1 restricting the process. Our dSTORM experiments revealed the striking homogenous distribution of AQP4 arrays at the PM of cultured cortical astrocytes. Over 67 000 clusters of AQP4 were examined, thus providing robust datasets for statistical analyses.

Single QD tracking has emerged as a powerful tool to study the movement of a variety of transmembrane molecules.^25,29–31^ Due to their photostability, brightness and relatively small size, the surface dynamics of QD-labeled molecules can be followed over a long period of time with a high spatial and temporal resolution. In high labeling density conditions, blinking patterns allowed us to determine that several QD moving together were labeling the same AQP4 array, confirming that QD and dSTORM strategies labeled the same structures.

To conclude, our study sheds new light on the regulation and functional organization of AQP4 in astrocytes, providing valuable insights into the way we understand CSF regulation in the brain. Our data suggest a new mechanism by which AQP4 mobility and cluster size are tuned by the adrenergic system and changes in extracellular tonicity, with strong implications for a molecular understanding of the regulation of sleep mediated solute clearance from the brain. Further studies are needed to explore effects of adrenergic signaling on the dystrophin-associated complex and extend our nanoscale characterization of AQP4 arrays to living brain slices and ultimately an *in vivo* scenario, where the architecture of astrocytic endfeet is fully functional and able to regulate CSF flow in the brain.

## Author contributions

ALZ and JAV designed experiments. ALZ, VM and NPH carried out experimental work. AHL assisted with single-particle tracking analysis. MHH led dSTORM imaging. ALZ and JAV wrote the manuscript. All authors read and approved the manuscript.

## Conflicts of interest

The authors declare no competing interests.

## Supplementary Material

NR-016-D4NR00330F-s001

NR-016-D4NR00330F-s002
