# Supplementary material for: Single-molecule imaging of aquaporin-4 array dynamics in astrocytes
Source: Nanoscale. 2024 Apr 29;16(19):9576–82. doi: 10.1039/d4nr00330f (PMC11097139; doi:10.1039/d4nr00330f)
Supplement: NR-016-D4NR00330F-s002 [file NR-016-D4NR00330F-s002.pdf]

Electronic Supplementary Material (ESI) for Nanoscale.
This journal is © The Royal Society of Chemistry 2024

# Supplementary Information

# Single-molecule imaging of Aquaporin-4 array dynamics in astrocytes

*Anna-Lena Zepernick[1], Vanya Metodieva[1], Noelia Pelegrina-Hidalgo[2,3,4], Anna H. Lippert[5], Mathew H. Horrocks[2,3] and Juan A. Varela[6]**

[1] School of Biology, University of St Andrews, St Andrews, UK.

[2] EaStCHEM School of Chemistry, The University of Edinburgh, Edinburgh, UK.

[3] IRR Chemistry Hub, Institute for Regeneration and Repair, The University of Edinburgh, Edinburgh, UK.

[4] Center for Regenerative Medicine, The University of Edinburgh, Edinburgh, UK.

[5] Institute for System Immunology, Julius-Maximilians-Universität Würzburg, Würzburg, Germany.

[6] School of Physics and Astronomy, University of St Andrews, St Andrews, UK.

* To whom correspondence should be addressed: jv32@st-andrews.ac.uk

## Table of Contents

*Materials and methods* ........ *3*

Primary cell culture ........ 3

Adeno-associated vectors and production ........ 3

Transduction ........ 6

Osmolarity and pharmacological treatment ........ 6

Immunocytochemistry in dissociated primary astrocytes ........ 6

AQP4 dSTORM and cluster analysis ........ 7

Quantum dot tracking in mixed cortical cultures ........ 9

Statistical analyses ........ 10

References ........ 11

*Supplementary Figures* ........ *12*

Supplementary Figure 1 ........ 12

Supplementary Figure 2 ........ 13

Supplementary Figure 3 ........ 14

Supplementary Figure 4 ........ 15

Supplementary Figure 5 ........ 16

Caption of Supplementary Movie ........ 17

## Materials and methods

### Primary cell culture

Mixed primary cortical cultures were obtained from postnatal 1-2 day old male and female C57BL/6J mice. The protocol was modified from Qui et al.[1] Briefly, pups were humanely sacrificed by cervical dislocation and decapitation in accordance with the UK Animals in Scientific Procedures Act (1986). Brains were placed in dissection solution [81.8 mM $Na_2SO_4$ (Sigma-Aldrich #S5640), 30 mM $K_2SO_4$ (Sigma-Aldrich #P9458), 5.84 mM $MgCl_2$ (Sigma-Aldrich #M8266), 0.252 mM $CaCl_2$ (Sigma-Aldrich #C1016), 1 mM HEPES (Sigma-Aldrich #H0887), 0.002 mM Phenol Red Solution (Sigma-Aldrich #P0290), 20 mM D-(+)-Glucose Solution (45%) (Sigma-Aldrich #G8769), with 0.05 mM Kynurenic acid (Sigma-Aldrich #K3375)]. Meninges were removed, cortical tissue was isolated and enzymatically digested [ 10 U/mL papain (Sigma-Aldrich #P4762) in dissection solution] for 2x20 minutes at 37°C, stirring every five minutes. The tissue was washed twice with dissection medium and twice with growth medium [Neurobasal-A-Medium (Gibco #12349015),1% Penicillin-Streptomycin (Gibco #15140122), 1x GlutaMAX™ (Gibco #35050061) and 1x B27 (Gibco #17504-044)]. Subsequently, cortical cells were dissociated in fresh growth medium and plated in 12-well-plates with 18 mm coverslips (VWR #631-0153P) pre-coated with 50 µg/mL poly-D-lysine (Sigma-Aldrich #P7886) at a density of 45 000 cells/$cm^2$. The primary mixed cultures were maintained at 37°C, humidified atmosphere of 5% CO2 and half of the culture medium was replaced every 48-72 hours until day *in vitro* (DIV) 8 and 9 when experiments took place.

### Adeno-associated vectors and production

*Vectors:*

To visualise astrocytes for quantum dot tracking, we utilised a targeted expression of green fluorescent protein (GFP) in astrocytes. This was achieved through AAV transduction of cytosolic (pAAV-GFAP-EGFP, gifted by Bryan Roth (Addgene plasmid # 50473 ; http://n2t.net/addgene:50473 ; RRID:Addgene_50473)) or membrane-bound EGFP (pAAV.GfaABC1D.PI.Lck-GFP.SV40, a gift from Baljit Khakh (Addgene plasmid # 105598 ; http://n2t.net/addgene:105598 ; RRID:Addgene_105598 -AAV5)) under an astrocyte-specific promoter. The production of AAV2/1 particles was achieved using pAAV2/1 which was a gift from James M. Wilson (Addgene plasmid # 112862 ; http://n2t.net/addgene:112862 ; RRID:Addgene_112862), and the helper plasmid pAdDeltaF6 which was a gift from James M. Wilson (Addgene plasmid # 112867 ; http://n2t.net/addgene:112867 ; RRID:Addgene_112867).

*AAV production:*

While pAAV.GfaABC1D.PI.Lck-GFP.SV40 was supplied as ready to use AAV5 particles, pAAV-GFAP-EGFP was produced and packaged into AAV2/1 particles in-house. The plasmid was supplied as a bacterial stab which was plated on agar plates (100 µg/ml ampicillin) and grown overnight at 37°C. Liquid bacterial cultures were grown 16-18 hours in a shaking incubator at 37°C, 180rpm. Bacteria were then pelleted at 4000x g, 4°C, 15 min (J26-XP, Beckman) after which ZymoPURE II Plasmid Maxi (Zymo Research, #D4203) was used for bulk DNA isolation by following manufacturer's instructions.

Human Embryonic Kidney 293T/17 (HEK293T/17) were obtained from ATCC and cultured in DMEM [(Fisher Scientific #31966021), supplemented with 10% foetal bovine serum (Gibco #26140079) and 1% PenStrep (Gibco, #15140122)] at 37°C, 5% CO2, humidified atmosphere. The growth medium was replaced every 3 days and cultures were passaged at 80% confluency using TrypLE (ThermoFisher, #12604-039). For AAV particle generation, the cells were seeded in sterile culture flasks (1125 $cm^2$ total

growth area) at a density of 115 000 cells per $cm^2$. The cultures were transfected 24-36 hours later using polyethylenimine (PEI) (VWR # 43896.03). The plasmid DNA was used at 1:1:1 molar ratio pAAV-GFAP-EGFP: pAdDeltaF6: and pAAV2/1. The DNA was diluted in 63 mL OptiMEM (FisherScientific #15392402) and combined with 2.6 mL PEI (1 mg/mL, pH 7.0). The solution was mixed, incubated at RT for 15 min, and combined with 124 mL low-serum DMEM (2% FBS). The final mix was used to replace the culture medium of the HEK293T/17.

*AAV purification:*

Following a 120-hour long incubation, the AAV-producing cells were harvested and AAV particles were isolated using Takara Maxi prep kit (Takara, #6678), following manufacturer's instruction. Briefly, the cells were detached through a 10 min incubation with 6.25 mM EDTA (pH 7.5, FisherScientific #10135423), pelleted by centrifugation at 2000x g for 10 min. The cell pellet was resuspended in AAV extraction solution A plus and incubated for 5 min at RT. Following a 10 min 5000g centrifugation at 4°C, the supernatant was treated with AAV Extraction solution B. Cryonase Cold-active Nuclease was added to the extract and incubated for 1 h at 37°C, after which Precipitator A was applied and incubated for 30min at 37°C. After the addition of Precipitator B, the mixture was vortexed and centrifuged at 5000x g for 5 min at 4°C. The supernatant was filtered through a Millex-HV 0.45 µm filter unit and transferred to an Amicon Ultra-4, 100kDa filter. The filter with the sample was centrifuged at 2000x g for 5 minutes at 15°C six times, adding fresh suspension buffer each time. The viral particles in the Amicon filter were resuspended and stored as single-use aliquots at -80°C. The titter of each viral prep was determined using AAV real-time PCR titration kit (Takara, #6233).

**Transduction**

Mixed cortical cultures were transduced at day *in vitro* 1 (DIV=1) by supplementing with fresh growth medium containing AAV particles (at 1 - 2.3*10^10 genome copies/mL). Half of the medium was replaced after 72 h and subsequent half medium changes were done every second day, until cultures reached DIV=9 and experimental treatments took place.

**Osmolarity and pharmacological treatment**

Osmolarities of all treatment solutions were measured using a vapor pressure osmometer (VAPRO® #5600). Cells were exposed to reductions in extracellular tonicity by diluting culture medium (246 mOsm) with sterile deionised water to 150 mOsm (hypotonic condition). An increase in extracellular tonicity from 246 mOsm (isotonic condition) to 370 mOsm (hypertonic condition) was achieved by sequential addition of 50 mM sodium chloride (Sigma-Aldrich # S5886) prepared in sterile deionised water.

We used isoproterenol hydrochloride (Sigma-Aldrich #I6504), a β-adrenergic agonist, to modulate adrenergic signalling in primary cortical cultures. Isoproterenol hydrochloride (ISO) was diluted to a 1 mM stock solution in sterile deionised water and kept at -80°C. Prior to experimental treatment, the agonist was diluted to a 1 µM final concentration in culture medium.

**Immunocytochemistry in dissociated primary astrocytes**

Cells were fixed in 1x PBS (Gibco #11510546) containing 4% paraformaldehyde (PFA) (Thermo Scientific™ # 11586711) and 4% sucrose (Sigma-Aldrich #S0389) for 10 min at 4°C under agitation (Grant-bio #PMR-30). After PBS wash the samples were permeabilized using 0.1% Triton-X-100 (Sigma-Aldrich #10789704001) in PBS for 10 minutes at 4°C under agitation. This step was omitted in case of cell surface staining of AQP4. The samples were then blocked in PBS supplemented with 5% bovine serum albumin

(BSA) (Sigma-Aldrich #A9647) for 45 minutes at 4°C under agitation. Primary antibodies were diluted in blocking solution and incubated for one hour at 4°C [rabbit anti-AQP4 (Sigma-Aldrich #HPA014784) 1:500, mouse anti-AQP4 (Sigma-Aldrich #MABN2526, clone E5415B) 1:200, and mouse anti-GFAP (Sigma-Aldrich #G3893, clone G-A-5) - 1:500]. Cells were washed with PBS at RT, and incubated with secondary antibodies diluted in blocking solution for 45 minutes at 4°C, protected from light [goat anti-rabbit Alexa Fluor® 647 (Invitrogen #A-21245) 1:1000, goat anti-mouse Alexa Fluor® 488 (Abcam #ab150117) 1:1000]. Cells were washed with PBS, mounted with anti-fade fluorescent mounting medium (Abcam #ab104135) onto microscope slides (VWR #631-1553), sealed with nail polish, and stored at 4°C protected from light until imaging.

**AQP4 dSTORM and cluster analysis**

For dSTORM experiments primary cultures (DIV = 9) grown on 18 mm coverslips were treated with 1 µM ISO or vehicle control for 1 h, immunostained as described above, and stored in PBS at 4°C in the dark until imaging.

Prior to imaging the coverslips were placed in a Ludin chamber containing 500 µL buffer [20 mM Trizma® hydrochloride solution (Sigma-Aldrich # T2194), 50 mM NaCl (Sigma-Aldrich #S5886), 10% glycerol (Thermo Scientific™ #106182940 and 10% glucose (Sigma-Aldrich #G6152), pH 7.5-8], supplemented with 60 µL reducing agent solution [1 M cysteamine (Sigma-Aldrich # M9768), pH 7.5-8] and 60 µL oxygen scavenging enzymes [57 µg/mL catalase from bovine liver (Sigma-Aldrich #C40) and 12 µg/mL pyranose oxidase from *coriolus sp*. (Sigma-Aldrich # P4234)]. All solutions were prepared in distilled water (Gibco™ #15230147), kept at -20°C and used within a week. TetraSpeck™ Microspheres of a nominal diameter of 0.1 µm, fluorescent blue/green/orange/dark red (Invitrogen #T7279) were added to the imaging medium at a 1:1000 dilution for drift correction.

The chamber was sealed off with a coverslip (VWR #631-0153P) to eliminate oxygen and thereby enhance fluorophore stability. Imaging was performed using a commercial TIRF microscope (Oxford Nanoimaging Ltd) fitted with an Olympus 1.4 NA 100x oil immersion super apochromatic objective and the laser illumination angle was set to 52 allowing for TIRF. Glial fibrillary acidic protein (GFAP) was initially imaged with standard GFP settings before proceeding with STORM imaging of AQP4. Sequential imaging and activation of Alexa Fluor 647 tagging AQP4 was done with a 630 nm laser (314 $W/cm^2$) and a 405 nm laser (212 $W/cm^2$), respectively. Streams of 200 images of Alexa Fluor 647 with an exposure time of 33 ms per frame were intercalated by activation pulses of 6.6 s with the 405 nm laser. These steps were repeated 40 times per field of view.

Resolution and localization number calculations were performed using the RustFRC python package as described previously[2] and the script is available at https://doi.org/10.5281/zenodo.7290477.

Custer analysis was performed using CODI, an Oxford Nanoimager developed software. To correct for potential sample drift during the acquisition, an inbuilt drift correction[3] was performed. Each localization was fitted to a 2D Gaussian distribution and any of those with a photon count lower than 5000 and or localization precision larger than 15 nm were discarded. Cluster analysis was done with density based spatial clustering of applications with noise (DBSCAN). Each cluster needed to have at least 15 localizations and each localization had to be within 150 nm of each other. The cluster area in $nm^2$ was plotted as a cumulative frequency, which allows to compare different sized samples and binning was set to 100 $nm^2$.

**Quantum dot tracking in mixed cortical cultures**

To determine the mobility of endogenous AQP4, the receptor was labelled with Quantum Dots (QD) similar to a previously described procedure[4]. 1 µM IgG (H+L) F(ab')2-Goat anti-Mouse, Qdot™ 655, Invitrogen™ (Fisher Scientific #Q-11021MP) were incubated with either 3 µg anti-AQP4 mouse (Sigma-Aldrich #MABN2526, clone E5415B) or 3 µg 6x-His tag mouse (Invitrogen #11533923, clone: HIS.H8) in 10 µL PBS for 30 minutes. To block unspecific binding, mouse serum (Abcam #ab7486, 10% final concentration) and goat serum (Sigma-Aldrich # G9023, 10% final concentration) were added to the pre-coated QD for at least 15 minutes and kept at 4°C, protected from light for up to 8 h. Cells were incubated with pre-coated QD (final dilution 0.1 nM) for 10 minutes at 37°C in imaging solution [growth medium supplemented with 10 mM HEPES (Sigma-Aldrich #H0887)] followed by three washes with imaging solution.

Cells were placed in a Ludin chamber and imaged at 37°C in a stage top incubator (Okolab #UNO-T-H-CO2) mounted onto a Nikon Eclipse Ti2 inverted microscope. The microscope was equipped with an ORCA-Flash4.0 V3 digital CMOS camera (HAMAMATSU #C13440-20CU) and a heated 100x/1.49 NA Apo TIRF objective (Nikon #CFI Apo TIRF 100XC oil). Transduced GFP-positive astrocytes were identified and imaged for ten consecutive frames and 50 ms exposure time using standard GFP imaging conditions. Subsequently, QD images were acquired for 500 consecutive frames with an exposure time of 50 ms using LED excitation filtered with a 531/40nm single-band bandpass filter (Semrock #FF01-531/40-25), a 562 nm standard epi-fluorescence dichroic beamsplitter (Semrock #FF562-DI03-25X36) and a 635 nm long-pass emission filter (Semrock #BLP01-635R-25). The microscope was controlled with Micro-Manager v2.0.

Streams of QD images were processed in Fiji[5] with the plugin TrackMate[6] over regions of interest (ROIs) imaged on GFP-positive astrocytes. A Laplacian of Gaussian (Log) filter and a median filter were used and sub-pixel localization were enabled. The detected maxima in the filtered image (first frame) were saved and used to calculate the number of QDs per $\mu m^2$. A simple linear assignment problem (LAP) tracker with a linking max distance of five pixels, gap-closing max distance of five pixels and gap-closing max frame gap of four was used. Trajectories were subsequently analysed in Matlab R2019b (Mathworks) to obtain the mean-squared displacement and instantaneous diffusion coefficients for each trajectory as previously described.[7] The minimal length of analysed tracks was set to 50 frames, and the diffusion coefficient of each track was calculated from linear fits of the first five points of the plot of mean squared displacement as a function of lag time over the trajectory. All diffusion coefficients were plotted as cumulative frequency distributions, with the binning set to 0.001 $\mu m^2/s$. Arrays were considered immobile when the diffusion coefficient was smaller than 0.001 $\mu m^2/s$. Fluorescence intensity over time of QD were obtained by tracking QD with the Spot Detection Tool of IMARIS 9.9.

**Statistical analyses**

Statistical analysis was performed on Prism 10.9.3 (GraphPad). To compare two individual groups, an unpaired, two-tailed t-test was used. However, if data did not pass the normality test (Shapiro-Wilk), we opted for Mann-Whitney test. Cumulative distributions were compared using Kolmogorov-Smirnov. A p-value below 0.05 was considered to be statistically significant.

## References


1 J. Qiu, O. Dando, P. S. Baxter, P. Hasel, S. Heron, T. I. Simpson and G. E. Hardingham, *Nature Protocols 2018 13:10*, 2018, **13**, 2176–2199.

2 H. Bhaskar, D. Kleinjan, C. Oi, Z. Gidden, S. J. Rosser, M. H. Horrocks and L. Regan, *Protein science: a publication of the Protein Society*, 2023, **32**, e4558.

3 J. Cnossen, T. J. Cui, C. Joo and C. Smith, *Optics express*, 2021, **29**, 27961–27974.

4 M. Heine, L. Groc, R. Frischknecht, J.-C. Béïque, B. Lounis, G. Rumbaugh, R. L. Huganir, L. Cognet and D. Choquet, *Science (New York, N.Y.)*, 2008, **320**, 201–5.

5 J. Schindelin, I. Arganda-Carreras, E. Frise, V. Kaynig, M. Longair, T. Pietzsch, S. Preibisch, C. Rueden, S. Saalfeld, B. Schmid, J.-Y. Tinevez, D. J. White, V. Hartenstein, K. Eliceiri, P. Tomancak and A. Cardona, *Nature Methods*, 2012, **9**, 676–682.

6 J.-Y. Tinevez, N. Perry, J. Schindelin, G. M. Hoopes, G. D. Reynolds, E. Laplantine, S. Y. Bednarek, S. L. Shorte and K. W. Eliceiri, *Methods*, 2017, 80–90.

7 L. Weimann, K. A. Ganzinger, J. McColl, K. L. Irvine, S. J. Davis, N. J. Gay, C. E. Bryant and D. Klenerman, *PLoS ONE*, 2013, **8**, e64287.

## Supplementary Figures

a)

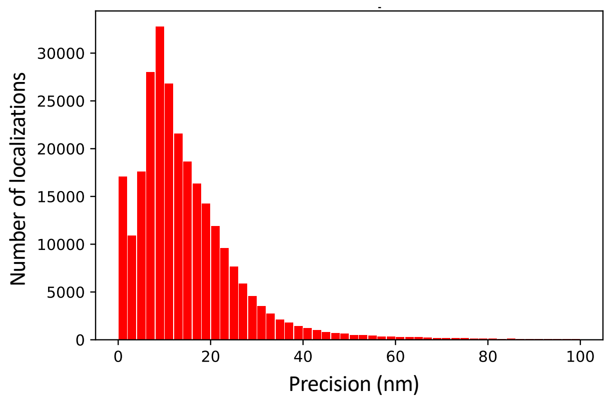


b)

Vehicle

| Precision (nm) | Resolution (nm) | Localizations |
|---|---|---|
| 14.81 | 41.32 | 264845 |
| 14.27 | 39.14 | 337174 |
| 13.23 | 33.18 | 688998 |
| 13.51 | 33.8 | 1319018 |
| 13.78 | 38.77 | 388329 |
| 16.63 | 40.45 | 642994 |
| 14.01 | 37.62 | 563018 |
| 15.06 | 40.29 | 848871 |

1 µM ISO

| Precision (nm) | Resolution (nm) | Localizations |
|---|---|---|
| 15.63 | 47.81 | 588415 |
| 13.66 | 36.94 | 488520 |
| 14.58 | 37.82 | 526661 |
| 17.46 | 49.43 | 307685 |
| 13.9 | 38.52 | 489297 |
| 12.7 | 39.37 | 273190 |
| 13.43 | 37.68 | 266194 |
| 16.99 | 47.4 | 367033 |
| 14.79 | 40.76 | 226333 |
| 15.38 | 43.02 | 515468 |
| 15.93 | 43.95 | 505486 |
| 16.27 | 46.22 | 483416 |

246 mOsm

| Precision (nm) | Resolution (nm) | Localizations |
|---|---|---|
| 18.11 | 48.59 | 777251 |
| 19.98 | 43.98 | 191556 |
| 14.71 | 39.89 | 205811 |
| 15.92 | 43.53 | 178440 |
| 22.22 | 59.33 | 507317 |
| 19.39 | 51.73 | 307318 |
| 21.53 | 60.22 | 581811 |
| 20.96 | 55.49 | 532151 |

370 mOsm

| Precision (nm) | Resolution (nm) | Localizations |
|---|---|---|
| 17.42 | 46 | 2058405 |
| 16.54 | 42.55 | 2071386 |
| 18.15 | 42.37 | 807438 |
| 17.43 | 44.04 | 1881970 |
| 18.13 | 44.37 | 1249206 |
| 16.53 | 45.5 | 408092 |
| 15.91 | 38.88 | 3107483 |
| 16.5 | 39.11 | 4554468 |

**Supplementary Figure 1**

**Localization precision. a)** Localization precision for all localizations in a full dataset corresponding to a biological replicate of all conditions (vehicle, adrenergic agonist, 246 mOsm and 370 mOsm) and **b)** the corresponding calculated precision and resolutions for the localizations of individual astrocytes in the dataset.

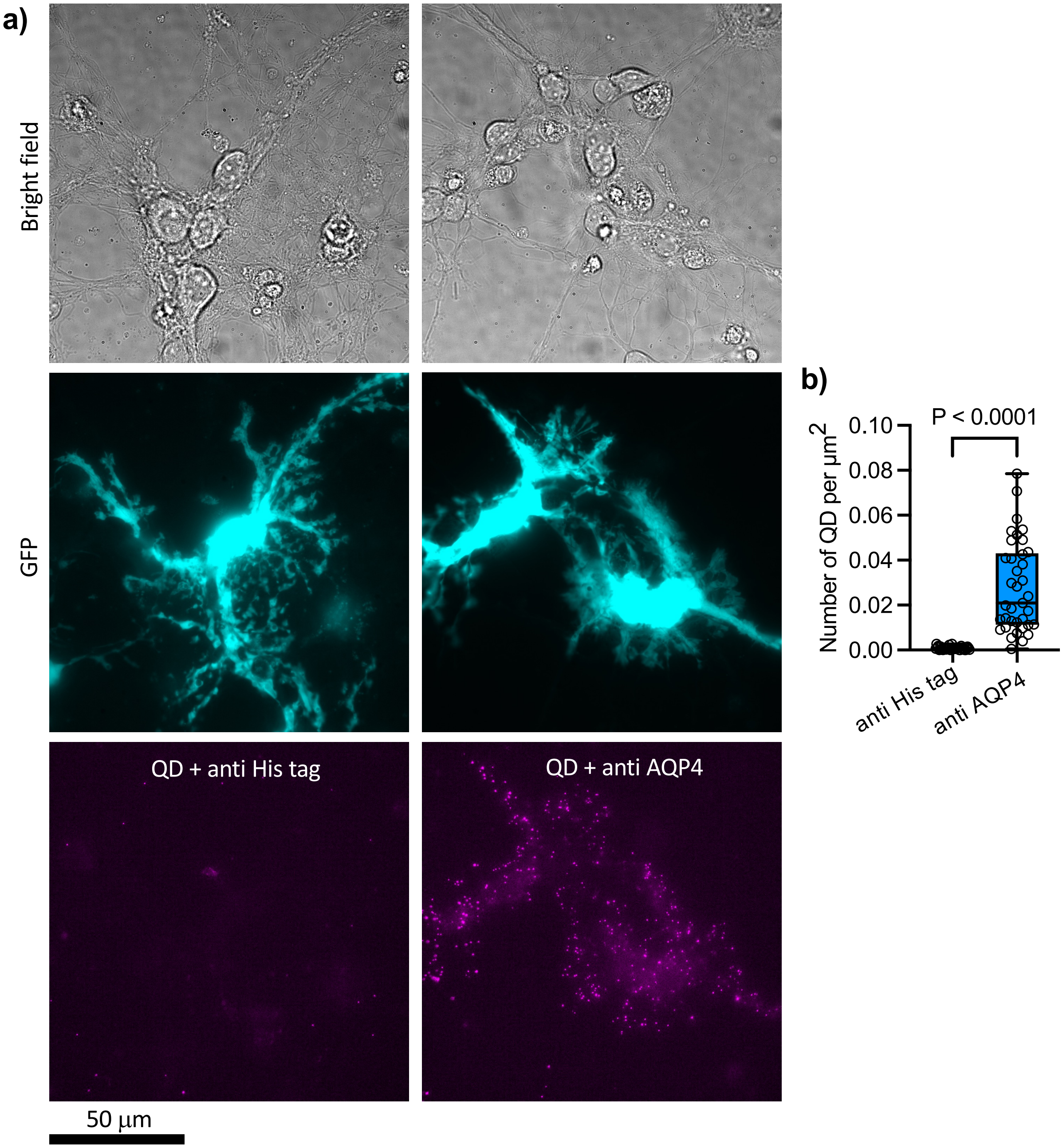


**Supplementary Figure 2**

**QD specificity and enrichment over astrocytes. a)** Images of mixed primary cultures showing Bright field, GFP signal and QD signal functionalized with an off-target antibody (left) and an anti AQP4 antibody. **b)** QD functionalized with anti-AQP4 antibody are significantly enriched over astrocytes transfected with cytosolic GFP compared to QD functionalized with anti-His tag antibodies.

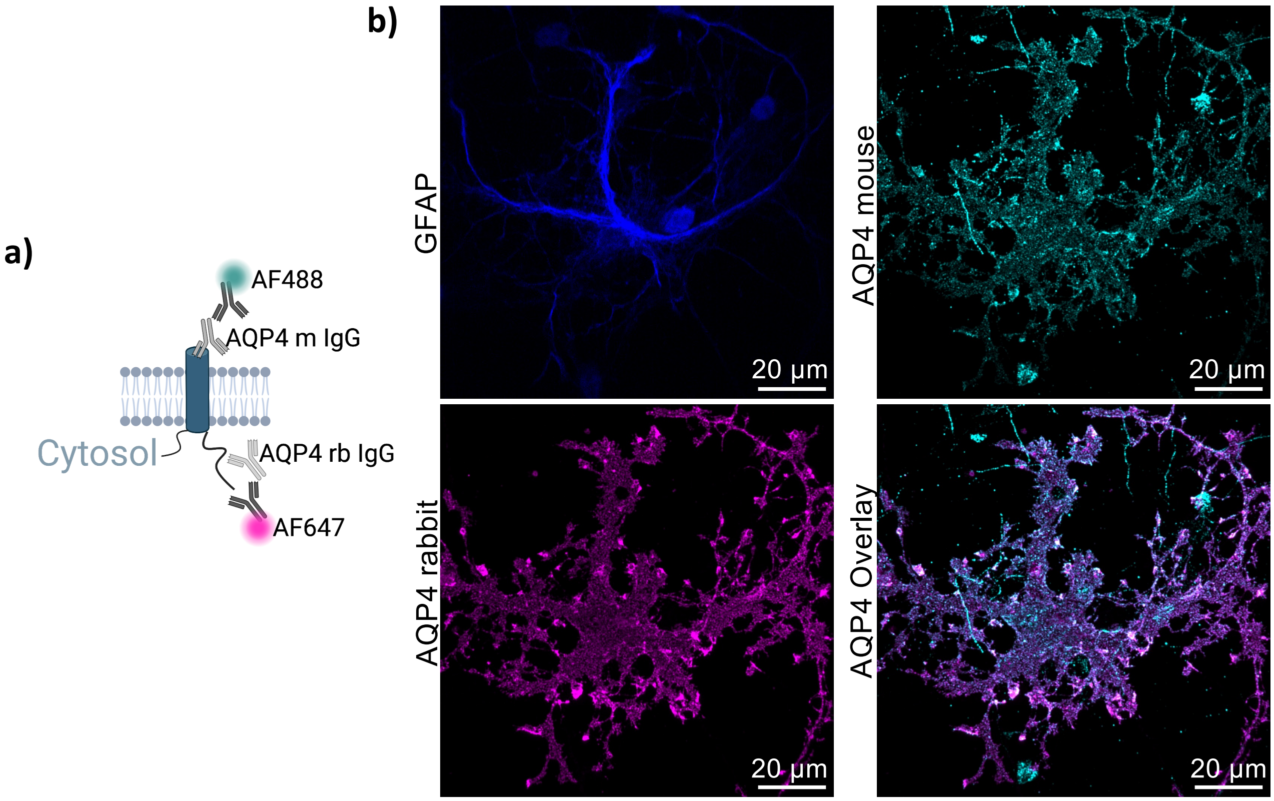


**Supplementary Figure 3**

**Validation of antibody staining for simultaneous QD and dSTORM imaging of AQP4. a)** Schematic of AQP4 double labelling with two different primary antibodies (raised in rabbit and mouse, targeting intracellular and extracellular epitopes, respectively). **b)** Representative spinning-disk confocal images of a GFAP-positive astrocyte indicating that the two anti-AQP4 antibodies co-localize.

Co-localization analysis:

- Pearson's Coefficient: r=0.671
- Overlap Coefficient: r=0.914

Manders' Coefficients of thresholded images:

- M1=0.526 (fraction of AQP4 rabbit overlapping AQP4 mouse)
- M2=0.767 (AQP4 mouse overlapping AQP4 rabbit)

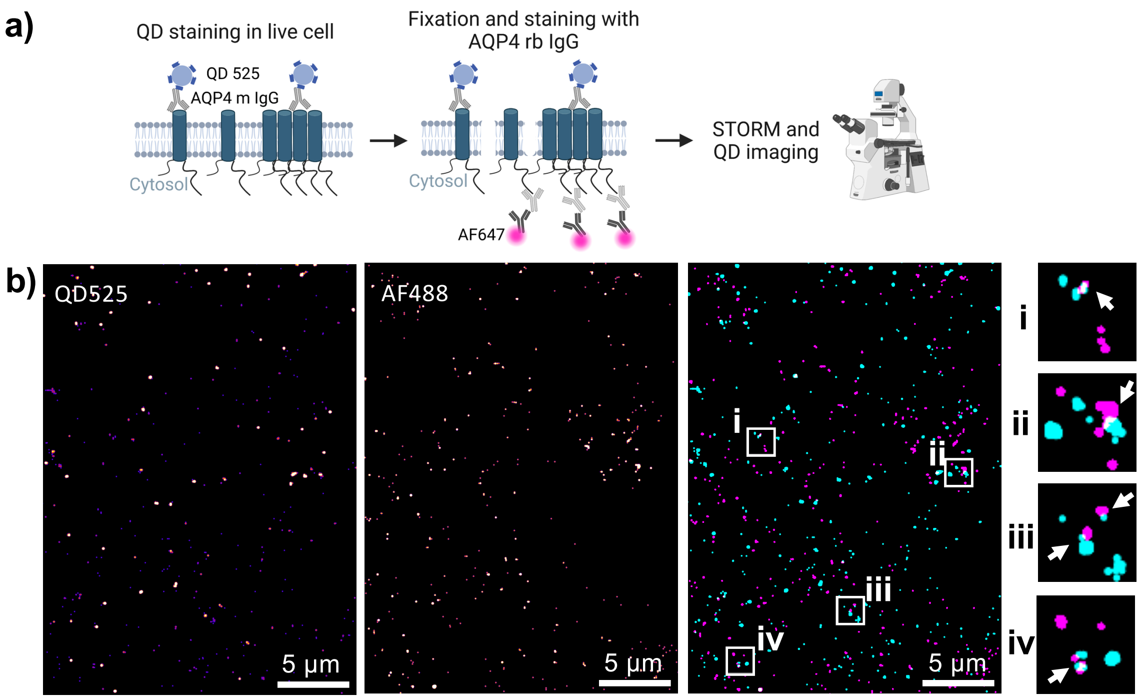


**Supplementary Figure 4**

**Simultaneous QD and dSTORM imaging of AQP4 in fixed astrocytes.** **a**) Experimental workflow for double labeling of AQP4 for single molecule imaging. Extracellular AQP4 was labeled with functionalized QDs *in vitro*, while intracellular AQP4 was tagged with antibodies functionalized with AF647 following fixation and permeabilization **b)** Representative images of astrocytes with double AQP4 labeling, reconstructed from TIRFM images of QD and AF647 in dSTORM conditions. Super-localized detections show AQP4 arrays co-labeled with both tags (zoom in sections of 2 µm x 2 µm, arrows show examples of AQP4 arrays co-labeled).

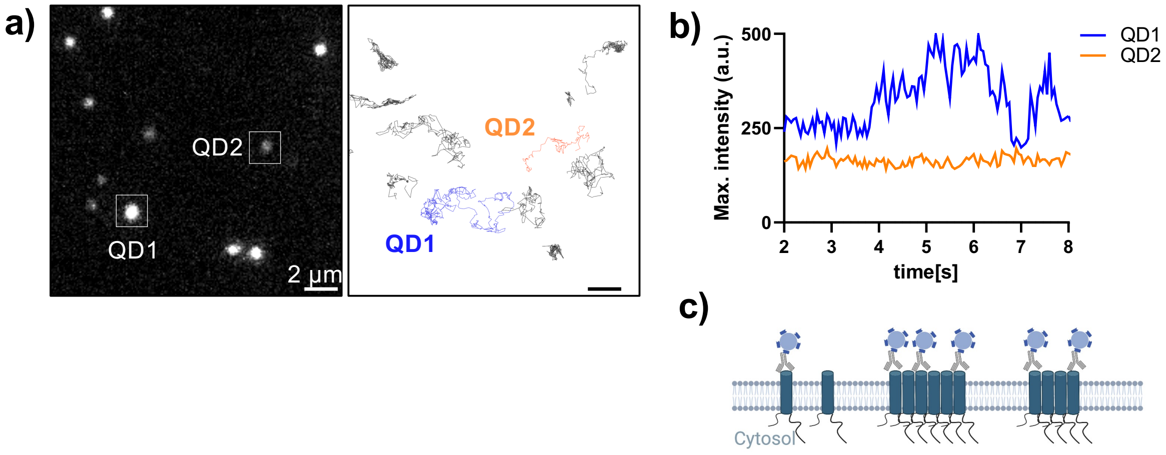


**Supplementary Figure 5**

**Multiple QDs can label the same AQP4 array. a)** A representative image of a cortical astrocyte with AQP4 labeled with high concentration of functionalised QDs, imaged at 20Hz sampling rate (left panel).The corresponding reconstructed trajectories are shown in the right panel. **b)** Intensity plots of two of the reconstructed trajectories showing that "QD1" are actually multiple QD, as indicated by the different intensity levels due to QD blinking behaviour. **c)** A schematic of AQP4 QD labelling, concluded from these high-density labelling experiments.

**Caption of Supplementary Movie**

Representative movie of QD labelled AQP4 in a primary astrocyte imaged at 20 Hz. QD shown in cyan, cytosolic GFP shown in orange to facilitate visualization.
